# Clinical Considerations of Isavuconazole Administration in High-Risk Hematological Patients: A Single-Center 5-Year Experience

**DOI:** 10.1007/s11046-021-00583-9

**Published:** 2021-08-25

**Authors:** Ilona Kronig, Stavroula Masouridi-Levrat, Yves Chalandon, Emmanouil Glampedakis, Nathalie Vernaz, Christian Van Delden, Dionysios Neofytos

**Affiliations:** 1 Division of Infectious Diseases, University Hospital of Geneva, Rue Gabrielle-Perret-Gentil 4, 1211 Geneva, Switzerland; 2 Bone Marrow Transplant Unit, Division of Hematology, Faculty of Medicine, University Hospital of Geneva, University of Geneva, Geneva, Switzerland; 3 Division of Infectious Diseases, University Hospital of Lausanne, Lausanne, Switzerland; 4 Geneva University Hospitals, University of Geneva, Geneva, Switzerland

**Keywords:** Isavuconazole, Prophylaxis, Treatment, Invasive mold infections, Acute myelogenous leukemia, Allogeneic hematopoietic cell transplant recipients

## Abstract

**Background:**

There are limited real-life data on isavuconazole prophylaxis and treatment of invasive mold infections (IMI) in hematological patients and allogeneic hematopoietic cell transplant (HCT) recipients.

**Objectives:**

Primary objective was to describe the indications of real-life isavuconazole administration at a university hospital. Secondary objectives included the description of liver function tests and QTc interval between baseline and end of treatment (EOT), clinical outcomes and breakthrough IMI by the EOT.

**Patients/Methods:**

This was a 5-year single-center retrospective study of all adult patients with acute myeloid leukemia and/or allogeneic HCT recipients who received isavuconazole as prophylaxis and/or treatment between June 1, 2016, and July 31, 2020.

**Results:**

Among 30 identified patients, the indications for isavuconazole administration were adverse events associated with prior antifungal treatment (*N*: 18, 60%: hepatotoxicity, renal insufficiency, long QTc interval, neurotoxicity, and potential drug–drug interactions in 6, 4, 3, 1 and 4 patients, respectively), clinical efficacy (*N*: 5, 16.6%), and other reasons (*N*: 10, 33.3%; 5/10 patients treated with isavuconazole to facilitate hospital discharge with orally administered appropriate treatment). Alanine aminotransferase significantly decreased from baseline (mean: 129 IU/L, range: 73, 202) to a mean of 48 IU/L (range: 20, 80) by day 14 (*P*-value: 0.02), 23.5 IU/L (range: 20, 27) by day 28 (*P*-value: 0.03) and 16.5 IU/L (range: 16, 17) by day 42 (*P*-value: 0.009). The QTc interval decreased from baseline (mean: 456.8 ms, range: 390, 533) to EOT (mean: 433.8 ms, range: 400, 472; *P*-value: 0.03). The mean isavuconazole plasma concentration was 2.9 mg/L (range: 0.9, 6.7). There was no breakthrough IMI observed.

**Conclusion:**

Isavuconazole is a safe and reliable antifungal agent in complex hematological patients, with relatively low hepatotoxicity and QTc interval shortening properties.

## Background

Isavuconazole is a second-generation triazole with a broad spectrum of activity against *Candida*, *Aspergillus* and most *Mucorales* spp., excellent adverse event profile, limited need for drug-level monitoring and established clinical efficacy [1–3]. It is currently approved for the treatment of invasive aspergillosis and mucormycosis [4–6]. Today, there are limited real-life data on isavuconazole use as primary and secondary prophylaxis and treatment of invasive mold infections (IMI) in hematological patients and allogeneic hematopoietic cell transplant (HCT) recipients [7–14]. Although the existing data suggest a good tolerability and safety profile, a variable rate of breakthrough IMI, ranging from 3 to 18%, has been observed [7, 11, 12, 14].

In this study, we review our 5-year experience with the administration of isavuconazole in high-risk hematology patients with acute myelogenous leukemia and/or allogeneic HCT recipients. We focused on pertinent questions associated with the use of isavuconazole in clinical practice, including the reasons for isavuconazole selection and effect of isavuconazole on liver function tests and QTc interval.

## Methods

This was a single-center retrospective observational cohort study to include all adult (≥ 18-year-old) patients with acute myeloid leukemia and/or allogeneic HCT recipients who received isavuconazole as prophylaxis and/or treatment between June 1, 2016 and July 31, 2020. In this real-life study, isavuconazole was used as primary treatment of invasive aspergillosis and other IMI, but also as primary antifungal prophylaxis and empirical antifungal treatment based on specific case requirements and as deemed appropriate by the clinical team. The study was approved by the local ethics committee (2020-01072). Patients with other than acute myeloid leukemia hematologic malignancies who did not receive an allogeneic HCT were not included.

### Study Objectives

The primary objective of this study was to describe the indications of real-life isavuconazole administration at a tertiary care center. The following secondary objectives were studied: (1) description of reasons for isavuconazole discontinuation, (2) distribution of liver function tests before and during isavuconazole administration, (3) assessment of QTc interval between baseline and end of treatment (EOT), and (4) clinical outcomes and breakthrough IMI by the EOT.

### Data Collection

Patients were retrospectively identified using the institutional HCT and pharmacy databases. The following data were collected using the institutional HCT database: (1) demographics, (2) underlying hematologic malignancy-related variables, including diagnosis, stage of disease, and administered treatments, and (3) pertinent HCT-associated variables, including conditioning regimen, HCT type and graft-versus-host disease (GvHD) grade ≥ 2. The following data were retrieved through chart review: (1) isavuconazole administration-related variables (indication for initiation and discontinuation, mode of administration, dose and duration), (2) laboratory values, including absolute neutrophil count (ANC), absolute lymphocyte count (ALC), alanine aminotransferase (ALT), gamma-glutamyl transferase (*γ*-GT), and total bilirubin 7 days prior to isavuconazole initiation (day 7), at baseline (day + 1) and on days + 7 (± 3 days), + 28 (± 3 days), + 42 (± 3 days), + 84 (± 3 days) and by EOT (± 7 days) of isavuconazole administration, (3) QTc interval at baseline and on days + 7 to + 14, + 28 to + 42 and by EOT (± 7 days) when available, and (4) administration of other than isavuconazole antifungal agents within 30 days prior and concomitantly with isavuconazole. Isavuconazole therapeutic drug monitoring (TDM) was performed as clinically indicated and relevant data were collected, when available.

### Definitions

Invasive fungal infections were defined based on established definitions by the European Organization for research and Treatment of Cancer/Mycoses Study Group [15]. Breakthrough IMI was defined as a probable/proven IMI diagnosed after a minimum of 7-day administration of appropriate antifungal treatment, as previously described [16, 17]. Clinical response was defined based on established guidelines [18]. Isavuconazole TDM was performed with liquid chromatography with tandem mass spectrometry (LC-MC/MC; RECIPE 200 Kit System, Germany). Empirical treatment was defined as administration of isavuconazole for the treatment of a suspicion of a possible IMI or in the setting of persistent neutropenic fever. Targeted antifungal treatment was defined as treatment of proven or probable IMI. Last follow-up day was considered the July 31, 2020, for those patients with ongoing isavuconazole administration. For these patients, EOT was considered to be the July 31, 2020, the day on which the database was sealed. Neutropenia was defined as an absolute neutrophil count < 500cells/mm^3^.

### Statistical Analysis

Standard descriptive statistics were used to summarize the study population characteristics. The Fisher’s exact or Chi-square tests were used for categorical variables and *t* test for continuous variables. Continuous variables were presented as means, with standard deviation and range. Statistical analysis was performed using STATA 16.0 (StataCorp, College Station, TX).

## Results

### Baseline Patient Characteristics

We identified 30 patients treated with isavuconazole at our institution during the study period (Table 1). The median age was 59 years (range: 18, 76), and 14 (46.7%) patients were female. The most frequently identified underlying hematologic malignancy was acute myeloid leukemia/myelodysplastic syndrome (24, 80%), and 18 (60%) patients received an allogeneic HCT. A total of 26 (86.7%) patients had received a chemotherapy regimen within the last 30 days before isavuconazole administration. Fourteen (46.6%) patients were neutropenic before isavuconazole initiation with a mean duration of neutropenia of 23.2 days (range: 8, 35).

**Table 1:** Baseline patient characteristics

Variables	Patients *N*: 30 (%)
Demographics	
Age (years), mean (range)	59 (18, 76)
Gender, female	14 (46.7)
Underlying hematologic malignancy	
Acute myeloid leukemia/myelodysplastic syndrome	24 (80)
Acute lymphoblastic leukemia	2 (6.6)
Lymphoma	2 (6.6)
Other^a^	2 (6.6)
Chemotherapy within 30 days prior to isavuconazole	26 (86.7)
Induction	11 (36.7)
Consolidation	9 (30)
Salvage	2 (6.7)
Conditioning for allogeneic HCT	4 (13.3)
Neutropenia prior to IVC administration**, **days Mean (range)	23.2 (8, 35)
HCT-associated variables^b^	18 (60)
Conditioning regimen	
Myeloablative	2 (11)
Reduced intensity	16 (88)
HCT donor	
HLA-matched related	5 (26.6)
HLA-matched unrelated	6 (33.3)
Haploidentical	7 (38.8)
HCT source	
Bone marrow	4 (22)
Peripheral blood stem cells	14 (78)
GvHD grade ≥ 2 before isavuconazole	6 (33)
Indication for isavuconazole administration	
Treatment	20 (77)
Proven IFI^c^	9 (45)
Probable IFI^d^	5 (25)
Possible IFI	6 (30)
Prophylaxis	10 (33)

*IVC* Isavuconazole, *HCT* hematopoietic cell transplant, *HLA* human leukocyte antigen, *GvHD* graft-versus host disease, *IFI* invasive fungal infection

^a^Other included: myeloid sarcoma (*N*: 1), multiple myeloma (*N*: 1)

^b^For all HCT-related variables, denominator included the total number of HCT recipients (*N*: 18)

^c^Proven IFI included 5 cases of invasive aspergillosis and 4 cases of mucormycosis

^d^Probable IFI included 5 cases of invasive aspergillosis

**Fig. 1:**
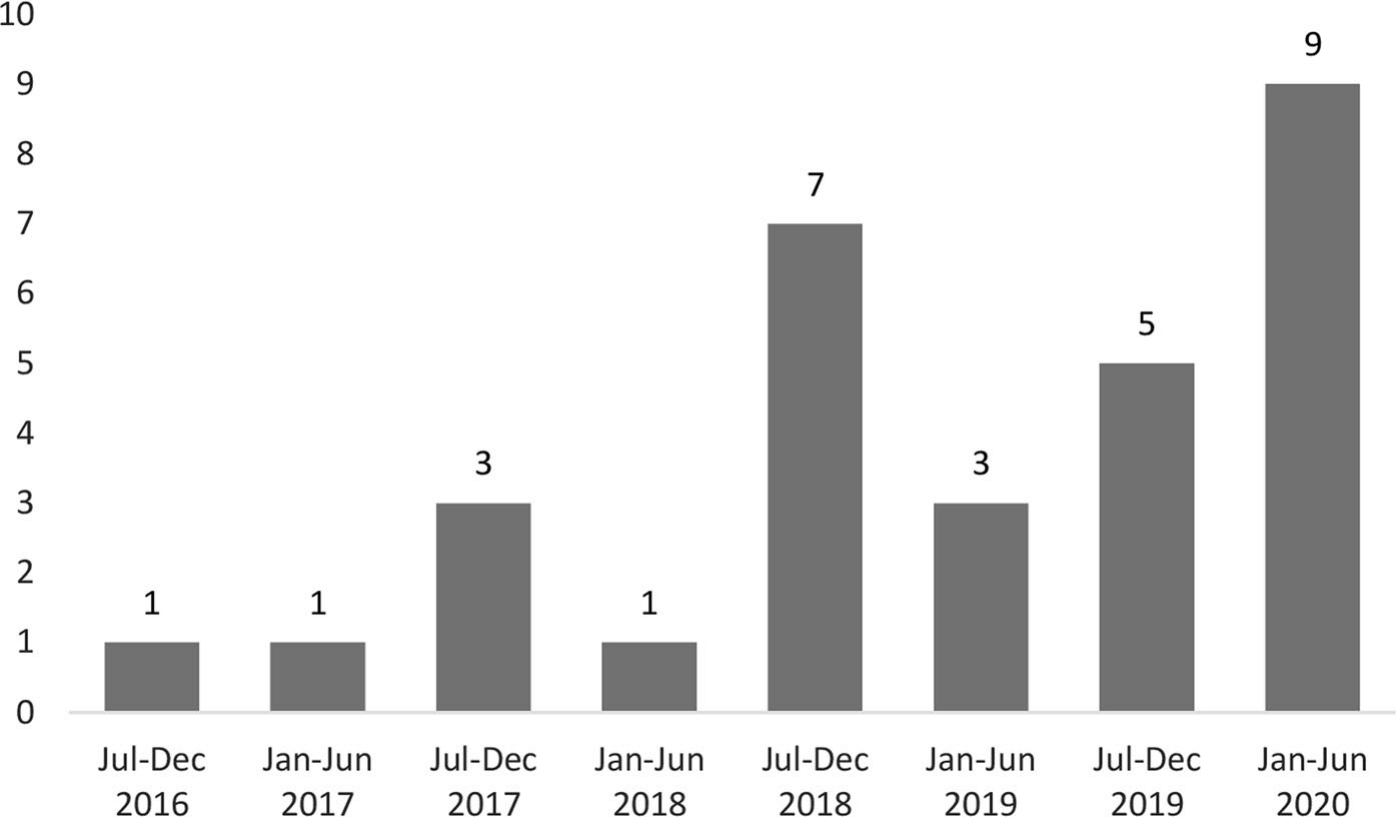
Administration of isavuconazole during the study period. The y-axis depicts the number of patients treated with isavuconazole per year

The distribution of isavuconazole administration during the study period is shown in Fig. 1. There was a significant increase in isavuconazole utilization during the second part of the study (July 2018 to July 2020) compared to the first part (June 2016–2018; *P*-value < 0.001). Isavuconazole was administered as primary prophylaxis in 10 (33.3%) patients and as treatment in 20 (66.6%) patients. Among 20 treated patients, 14 (70%) and 6 (30%) patients received isavuconazole as targeted and empirical therapy, respectively. Treatment was administered for a proven, probable and possible IMI in 9 (45%), 5 (25%) and 6 (30%) patients, respectively (Table 2).

**Table 2:** Detailed description of 20 patients, who were treated with isavuconazole for a proven, probable or possible invasive mold infection

Age	Gender	Heme Malignancy	Chemotherapy 30 days before IVC	Antifungal 30 days before IVC	Allo- HCT	GvHD
76	Male	AML	Induction	PCZ, ECH		
68	Male	MDS	Consolidation	FCZ	Yes	No
51	Female	AML	Induction	L-AMB		
41	Female	MDS	Consolidation	PCZ	Yes	No
42	Male	AML	Induction	L-AMB		
62	Female	AML	Induction	L-AMB		
61	Male	ALL	Consolidation	L-AMB + ECH + VCZ	Yes	Yes
65	Male	AML	Consolidation	L-AMB	Yes	Yes
50	Female	MDS	Consolidation	PCZ	Yes	No
60	Male	AML	Salvage	PCZ	Yes	No
67	Male	AML	Induction	VCZ		
70	Female	AML	Induction	VCZ		
74	Male	AML	Induction	PCZ	Yes	No
17	Male	ALL	Consolidation	ECH	Yes	No
59	Female	Lymphoma	None	L-AMB	Yes	No
58	Male	MDS	None	PCZ, ECH	Yes	Yes
74	Female	MDS	Salvage	L-AMB + ECH		
69	Male	AML	Induction	FCZ	Yes	No
43	Male	AML	Conditioning	L-AMB	Yes	Yes
53	Male	Lymphoma	Conditioning	L-AMB	Yes	No

Age	Gender	Neutropenia days before IVC	Neutropenia days after IVC	Treatment Type	IFI	Reason for IVC start	Reason of IVC stop	IVC days
76	Male	26	0	Targeted	Proven IA	Hepatotoxicity	Death	124
68	Male	21	4	Empirical	Possible IA	Renal insufficiency	No IFI	7
51	Female	19	30	Empirical	Possible IA	Simplification	AE (rash)	2
41	Female	0	0	Targeted	Proven IA	Neurotoxicity	Cost	9
42	Male	13	10	Targeted	Probable IA	Hepatotoxicity	Simplification	6
62	Female	8	15	Targeted	Probable IA	Simplification	Hematological disease progression	11
61	Male	0	0	Targeted	Proven mucormycosis	Better CNS penetration	AE (rash)	1
65	Male	0	0	Empirical	Possible IA	Hepatotoxicity, low PCZ level	Simplification	81
50	Female	0	0	Targeted	Proven IA	Long QTc	Death	4
60	Male	0	15	Targeted	Probable IA	Empirical	Hematological disease progression	20
67	Male	0	0	Targeted	Proven mucormycosis	Combination treatment	Ongoing	568
70	Female	0	0	Targeted	Probable IA	Hepatotoxicity	End of treatment	257
74	Male	30	10	Empirical	Possible IA	Renal insufficiency	Simplification	69
17	Male	0	0	Targeted	Proven IA	Simplification	End of treatment	270
59	Female	0	0	Empirical	Possible IA	Renal insufficiency	Cost	36
58	Male	0	0	Targeted	Proven cryptococcosis	Hepatotoxicity	Cost	127
74	Female	10	7	Targeted	Proven Mucormycosis	Combination treatment	Ongoing	151
69	Male	0	0	Targeted	Probable IA	Empirical	Hematological disease progression	165
43	Male	0	0	Targeted	Proven IA	Simplification	Ongoing	170
53	Male	30	0	Empirical	Possible IA	Simplification	No IFI	10

*IVC* Isavuconazole, *HCT* hematopoietic cell transplant, *GvHD* graft versus host disease, *IFI* invasive fungal infection, *AML* acute myelogenous leukemia, *POS* posaconazole, *ECH* echinocandin, *IA* invasive aspergillosis, *MDS* myelodysplastic syndrome, *FCZ*: fluconazole, *L-AMB* liposomal amphotericin-B, *AE* adverse event, *VCZ* voriconazole, *ALL* acute lymphoblastic leukemia, *CNS* central nervous system

### Isavuconazole Administration Data

All patients received a loading dose of isavuconazole and 200 mg once daily as regular dose (Table 3). Mean duration of isavuconazole administration was 87.6 days (range: 1, 568), 12.2 days (range: 1, 127) and 117.3 days (range: 1, 568) for intravenous and oral administration, respectively. The vast majority of patients (*N*: 27, 90%) had received another antifungal agent within 30 days prior to isavuconazole, including posaconazole (*N*: 7, either intravenously or as an extended release oral formulation), liposomal amphotericin-B (*N*: 6), fluconazole (*N*: 6), voriconazole (*N*: 2), and an echinocandin (*N*: 5).

**Table 3:** Data on isavuconazole administration

Variables	Patients *N*: 30 (%)
Dose	
Loading dose 200 mg 3 times daily for 2 days	30 (100)
Maintenance dose 200 mg once daily	30 (100)
Formulation	
Intravenous only	10 (33)
Orally only	10 (33)
Intravenous followed by orally	10 (33)
Duration	
Overall duration, days mean (range)	87.6 (1, 568)
Intravenous administration duration, days mean (range)	12.2 (1, 127)
Oral administration duration, days mean (range)	117.3 (1, 568)
Prior administration of other antifungal agents^a^	27 (90)
Posaconazole	7
Liposomal amphotericin-B	6
Fluconazole	6
Voriconazole	2
Echinocandin	5
No antifungal	3
Concomitant administration with other antifungal agents	1 (3.3)
Neutropenia during IVC administration, days mean (range)	18.8 (4, 30)
Indication for IVC administration^b^	
Adverse events of prior treatment	14(46.6)
Hepatotoxicity	6
Renal insufficiency	4
Long QTc interval	3
Central nervous system	1
Potential drug–drug interactions	4 (13.3%)
Clinical efficacy	5 (16.6)
Sub-therapeutic posaconazole level	1
Combination treatment	4
Other^c^	10 (33.3)
Indication for IVC discontinuation^b^	
Treatment completion	5 (16.7)
Treatment de-escalation	5 (16.7)
Adverse events	5 (16.7)
Rash	2
Hepatotoxicity	2
Drug–drug interactions	1
Insurance coverage	3 (10)
Death	3 (10)
Disease progression	3 (10)
Ongoing^d^	3 (10)

*IVC* Isavuconazole, *IFI* invasive fungal infection

^a^Administration of other antifungal agents during the 30 days prior to isavuconazole administration. A patient could have received more than one antifungal agent prior to isavuconazole administration

^b^A patient could have more than one indication for isavuconazole administration and/or discontinuation

^c^Other reasons for initiation of treatment with isavuconazole included the following: (1) transition from intravenous to orally administered treatment in 5 patients, (2) treating team choice for two cases of probable pulmonary invasive aspergillosis and a patient with proven pulmonary and cerebral *Rhizomucor pusillus* mucormycosis, (3) no specific indication in two patients

^d^Three patients were still on isavuconazole at the end of the study period

### Reasons for Isavuconazole Initiation

Patients received isavuconazole for: (1) adverse events and potential drug–drug interactions associated with the administration of prior antifungal treatment (*N*: 18, 60%), (2) clinical efficacy (*N*: 5, 16.6%), and (3) other reasons (*N*: 10, 33.3%). Among patients with adverse events associated with prior antifungal agents, isavuconazole was started due to elevated liver tests in 6 patients, underlying renal insufficiency in 4 patients, long QTc interval in 3 patients, underlying central nervous system toxicity in 1 patient, and potential interactions between voriconazole or posaconazole and chemotherapy in 4 patients. In 5 patients requiring administration of isavuconazole for clinical efficacy reasons, the selection of isavuconazole was based on sub-therapeutic drug levels of posaconazole in 1 patient and in severe IMI requiring combination treatment in 4 patients: (1) isavuconazole with an echinocandin for the treatment of a probable IMI due to *Aspergillus terreus*, (2) isavuconazole with an echinocandin in a patient with severe neutropenic enterocolitis for broader anti-*Candida* coverage, (3) isavuconazole with liposomal amphotericin-B for the treatment of a proven pulmonary IMI due to *Rhizomucor* and (4) isavuconazole with liposomal amphotericin-B and an echinocandin for the treatment of a disseminated *Rhizomucor* infection. In the rest of cases, isavuconazole was used for treatment simplification in 5 patients, replacing liposomal amphotericin-B (*N*: 3) and an echinocandin (*N*: 2), to facilitate hospital discharge with an orally administered appropriate treatment. Finally, isavuconazole was used as primary antifungal treatment in two cases of probable pulmonary aspergillosis and one patient with proven pulmonary and cerebral mucormycosis, respectively. In two patients, the reason for isavuconazole selection was not documented in the patient charts.

### Reasons for Isavuconazole Discontinuation

Isavuconazole treatment was discontinued prematurely in 5 (16%) patients due to: (1) type-I drug hypersensitivity reaction (*N*: 2), (2) elevated liver enzymes (*N*: 2) and (3) a potential drug–drug interaction with a chemotherapy treatment (*N*: 1). Type-I drug hypersensitivity reaction was suspected in a patient who developed a maculopapular rash, nausea and vomiting after 24 h of isavuconazole treatment initiation and in one patient with a maculopapular rash and severe cardiothoracic pain with elevated cardiac enzymes just after the first infusion. The latter was subsequently attributed to administration of isavuconazole intravenously without using the specific filter required for administration. Although an isavuconazole-related allergic reaction was not confirmed, isavuconazole treatment was not re-initiated in any of these two patients. Among the two patients whose isavuconazole was discontinued due to moderate liver function abnormalities, one had received chemotherapy within 48 h prior and in the second patient isavuconazole was co-administered with several other potentially hepatotoxic agents.

### Distribution of Liver Function Tests During Isavuconazole Administration

**Fig. 2:**
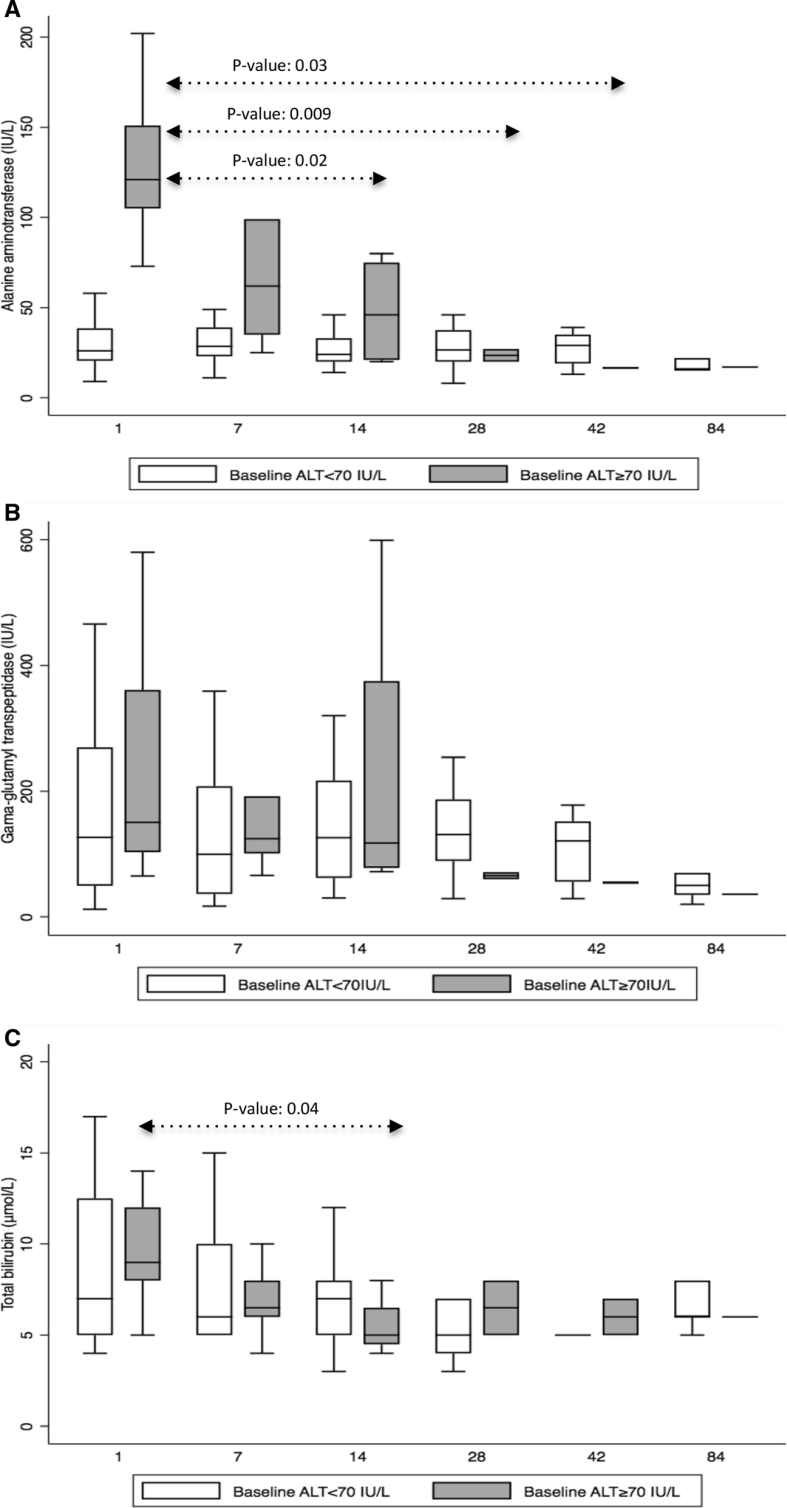
Distribution of (**a**) alanine aminotransferase (ALT; IU/L), (**b**) gamma-glutamyl transferase (*γ*-GT; IU/L), and (**c**) total bilirubin (µmol/L) at baseline (day + 1) and on days + 7(± 3 days), + 14(± 3 days), + 28(± 3 days), + 42(± 3 days), and + 84(± 3 days) of isavuconazole administration presented in box plots, for patients with and without baseline hepatotoxicity, defined as ALT ≥ 70 IU/L. Boxes represent the median and 25th and 75th percentiles, whiskers represent the range of maximum and minimum values within the interquartile range. Outliers are not shown. *P*-values are presented only for statistically significant differences. The X-axis represents days of isavuconazole administration: baseline (day + 1) and days +7(± 3 days), + 14(± 3 days), + 28(± 3 days), + 42(± 3 days), and + 84(± 3 days)

The mean ALT value at baseline was 49.3 IU/L (range: 9, 202). As isavuconazole was administered in 6 (20%) patients due to liver function test abnormalities associated with prior antifungal treatments, we divided patients in two groups: those 6 (20%) patients with prior hepatotoxicity (baseline ALT ≥ 70 IU/L) and 24 (80%) patients without baseline liver function abnormalities (baseline ALT < 70 IU/L). We analyzed the distribution of ALT, *γ*-GT and total bilirubin during the first 84 days of isavuconazole administration between the two groups (Fig. 2). In the baseline-hepatotoxicity group, ALT decreased from a mean of 129 IU/L (range: 73, 202) at baseline to 106.5 IU/L (range: 25, 356) by day + 7 (*P*-value: 0.58), 48 IU/L (range: 20, 80) by day + 14 (*P*-value: 0.02), 23.5 IU/L (range: 20, 27) by day + 28 (*P*-value: 0.03), 16.5 IU/L (range: 16, 17) by day + 42 (*P*-value: 0.009) and 17 IU/L (range: 17, 17) by day + 84 (*P*-value: not available, only one patient; Fig. 2a). There were no significant differences in *γ*-GT mean values between baseline and day + 84 of isavuconazole administration in the hepatotoxicity group (Fig. 2b). Total bilirubin decreased from a mean of 9.5 μmol/L (range: 5, 14) at baseline to 6.8 μmol/L (range: 4, 10) by day 7 (*P*-value: 0.08), 5.5 μmol/L (range: 4, 8) by day 14 (*P*-value: 0.04), 6.5 μmol/L (range: 5, 8) by day + 28 (*P*-value: 0.23), 6 μmol/L (range: 5, 7) by day + 42 (*P*-value: 0.18) and 6 μmol/L (range 6, 6) by day + 84 (*P*-value: not available, only one patient; Fig. 2c). Among 24 patients with baseline ALT < 70 IU/L, ALT, *γ*-GT and total bilirubin remained stable from baseline to day + 84 of isavuconazole administration without significant differences (data not shown).

### Description of QTc Interval During Isavuconazole Administration

**Fig. 3:**
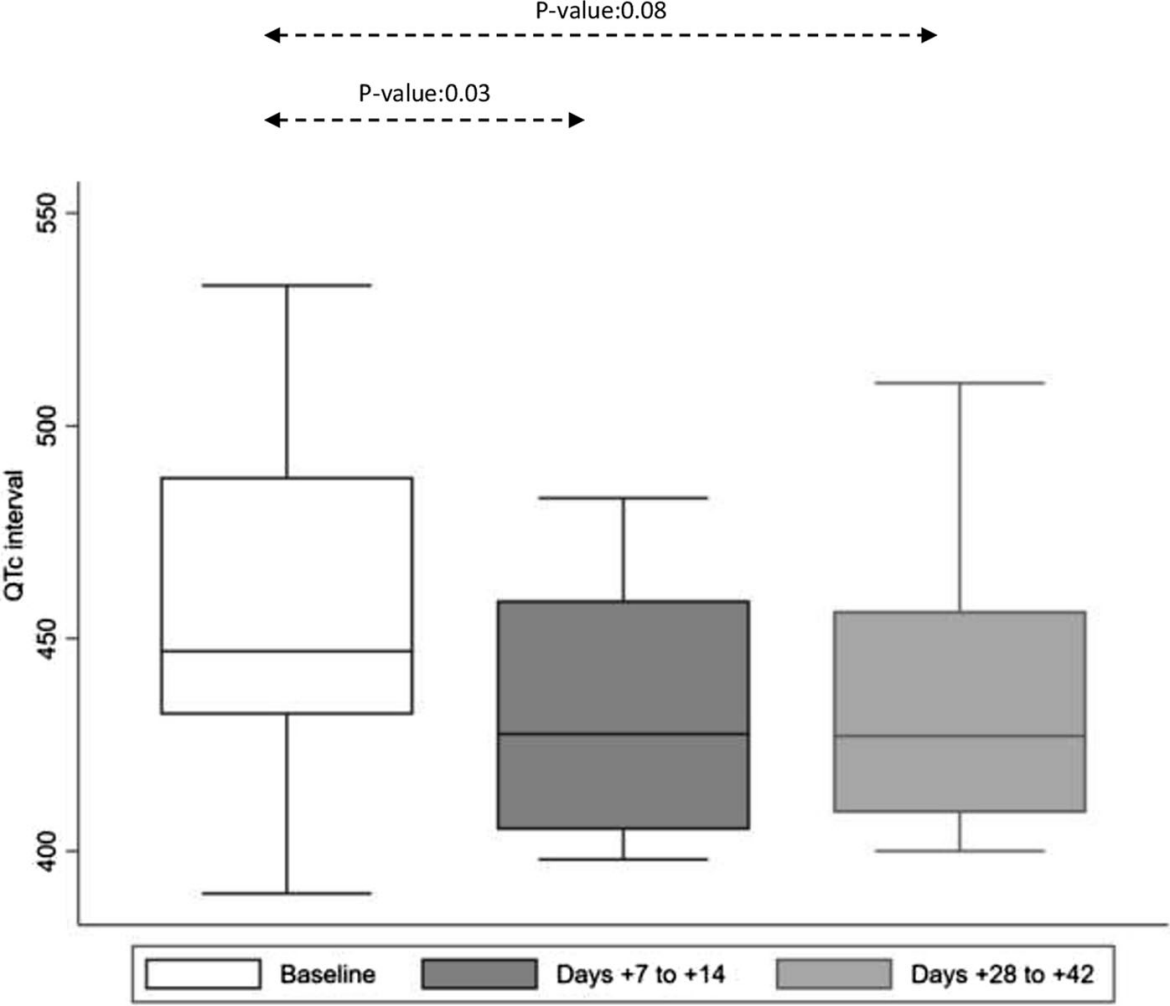
Distribution of QTc interval in msec at baseline and during isavuconazole administration presented as box plots. QTc interval measurement was available for 29 patients at baseline. Due to the fact that QTc interval measurements were not available at all time points during the study period, we included QTc between 7 and 14 (14 patients) and 28–42 days (12 patients) of isavuconazole administration. There was a significant decrease in QTc interval from baseline to days 7–14 and a trend for shorter QTc interval between days 28–42 of isavuconazole administration. Boxes represent the median and 25th and 75th percentiles; whiskers represent the range of maximum and minimum values within the interquartile range

A total of 29 patients had available QTc data at baseline, with a mean QTc interval at 456.8 ms (range: 390, 533; Fig. 3). Among the 12 patients with available QTc interval by the EOT, the mean QTc was at 433.8 ms (range: 400, 472), significantly shorter than at baseline (*P*-value: 0.03). Fourteen patients had available QTc interval data between days 7–14 of isavuconazole administration, with a mean of 432.2 ms (range: 398, 483), significantly shorter from baseline (*P*-value: 0.03). There was a trend for shorter QTc interval between baseline and days 28–42 (mean: 435.4, range: 400, 510; *P*-value: 0.08).

### Isavuconazole TDM

**Fig. 4:**
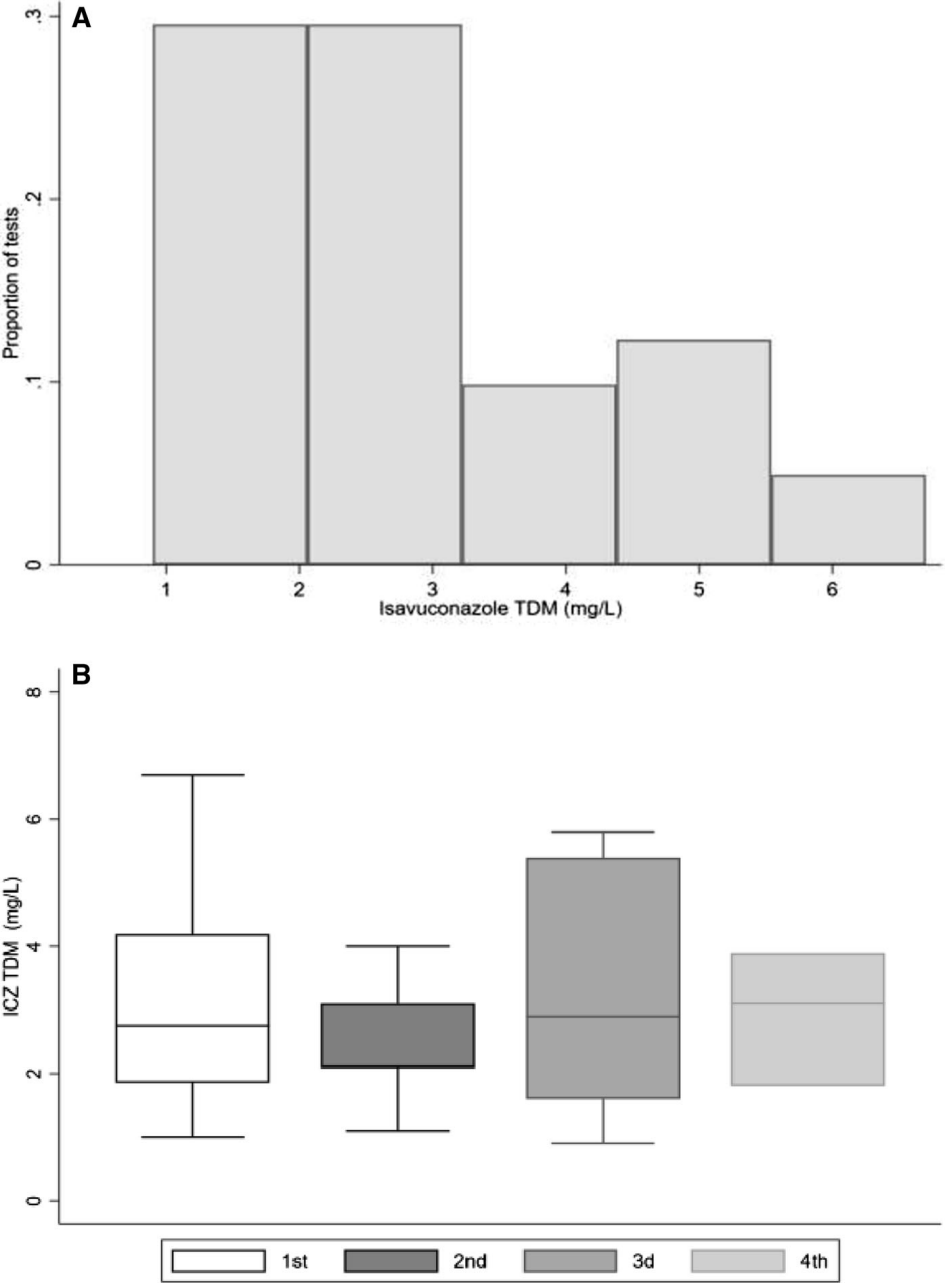
**a** Therapeutic drug monitoring isavuconazole concentrations (mg/L) presented as a histogram. All isavuconazole measurements are included. **b** Isavuconazole plasma concentrations (mg/L) presented as box plots for 16, 9, 7 and 3 patients who had 1, 2, 3 and 4 different isavuconazole concentration measurements, respectively. Boxes represent the median and 25th and 75th percentiles; whiskers represent the range of maximum and minimum values within the interquartile range. Outliers are not shown

A total of 35 TDM tests were performed in 16 (43.3%) patients (median: 1 test/patient, range: 0–4). The mean isavuconazole concentration was 2.9 mg/L (range: 0.9, 6.7; Fig. 4a). Seven patients had only one isavuconazole TDM, while 2, 4 and 3 patients had 2, 3 and 4 TDM tests performed, respectively. The first TDM was performed at a median of 9 days (range: 3, 168) after isavuconazole initiation. The mean concentration of isavuconazole was 3.0 mg/L (range: 1, 6.7), 2.5 mg/L (range: 1.1, 4.0), 3.3 mg/L (range: 0.9, 5.8) and 2.9 mg/L (range: 1.8, 3.9) on the 1st, 2nd, 3rd and 4th measurements, respectively (Fig. 4b). There was no statistically significant difference between the four different measurements of isavuconazole concentrations.

### Clinical Outcomes

None of the 30 patients receiving isavuconazole developed a breakthrough IMI during the study period. Three (10%) patients died due to uncontrolled underlying malignancy. None of the patients included died due to an IMI.

## Discussion

This 5-year single-center retrospective cohort study further expands our insight of the major clinical considerations associated with the use of isavuconazole in a real-life setting. We detail the reasons leading to the selection of this agent, its effect on liver function and cardiac rhythm, and a stable and reliable plasma concentrations over time.

Selecting the appropriate molecule or orally administered treatment sequence for the prophylaxis or treatment of IMI in complex poly-medicated and fragile hematological patients and/or allogeneic HCT recipients remains a major challenge. A large number of variables need to be considered, including, but not limited to, possible adverse events, drug–drug interactions, tolerability, bioavailability, absorption and availability of orally administered treatment. Nevertheless, antifungal agent administration is frequently interrupted, for instance voriconazole prophylaxis is discontinued in almost 50% of allogeneic HCT recipients receiving this agent [19, 20]. The need for safer, better tolerated antifungal treatment was the driving force for isavuconazole selection in the vast majority of cases in our study, due to adverse events or drug–drug interactions associated with a previously administered antifungal treatment. A large number of treatments administered in hematologic malignancy patients, including chemotherapy agents, are metabolized through the cytochrome P-450 (CYP) pathway, with potential important interactions with agents, such as voriconazole and posaconazole, both strong inhibitors of CYP3A4. Isavuconazole being a modest CYP3A4 inhibitor has fewer significant interactions allowing easier co-administration with other agents [1]. Well-known azole-related toxicities, including hepatotoxicity and prolongation of QTc interval, were the driving reasons leading to the replacement of another azole by isavuconazole in this study.

Indeed, isavuconazole appears to be less hepatotoxic when compared to other azoles. In the pivotal validation clinical trial, isavuconazole was associated with significantly less frequent liver test abnormalities, when compared to voriconazole [2]. Similarly, in a cohort of high-risk patients with hematologic malignancy from MD Anderson treated with posaconazole, liver tests rapidly dropped after transition from posaconazole to isavuconazole [9]. Consistent with prior reports and despite the small number of patients, our data suggest that isavuconazole may be safely used, even in patients with abnormal baseline liver function with rapid decline of transaminases within the first two weeks after isavuconazole initiation. Notably, the degree of baseline hepatotoxicity was relatively low, with a mean ALT in the “baseline hepatotoxicity” group of 129 IU/L. Furthermore, other interventions, such as discontinuation of other potentially hepatotoxic agents, which could have happened concomitantly with the introduction of isavuconazole, were not considered. More data are required to further study the safety and effect of isavuconazole on liver function in high-risk patients.

Hematology malignancy patients and allogeneic HCT recipients frequently require co-administration of more than one agents with potential QTc-prolonging effect (e.g., broad-spectrum azoles, macrolides, fluoroquinolones, chemotherapy agents, etc.). The unique characteristic of isavuconazole of shortening the QTc interval may allow clinicians to use this agent in patients with prolonged QTc interval, in whom an azole would have been previously avoided. Currently, there are few data on the beneficial effect of isavuconazole on the QTc interval [21]. Our data demonstrate a clear and robust decrease in the QTc interval under treatment with isavuconazole, which can be of use in specific cases.

Treating severe IMI, particularly those due to *Mucorales* spp., frequently requires administration of intravenously administered amphotericin-B as a first-line therapy [6, 22]. With more patients infected by angioinvasive molds surviving and eventually being discharged from the hospital, transition to an orally administered, effective and well-tolerated treatment is frequently required in clinical practice. Isavuconazole was used in almost one-third of patients in this series as a transition to an orally administered treatment to facilitate hospital discharge. Considering the excellent bioavailability, reliable pharmacokinetics and plasma concentrations and broad-spectrum activity of this agent, isavuconazole appears to be a desirable option for a step-down orally administered treatment of IMI high-risk patients [6].

As previously demonstrated, the rate of breakthrough IMI in patients treated with isavuconazole is variable, ranging from 3 to 18% [8, 11, 12, 14]. Breakthrough IMI has been mostly due to non-*Aspergillus* molds infections or non-*albicans Candida* invasive candidiasis, predominately reported in patients who had received prior antifungal treatments, with relapsed/refractory leukemia and prolonged neutropenia at the time of isavuconazole introduction. None of our patients receiving isavuconazole developed a b-IMI during the study period. This may be, in part, due to the small numbers of high-risk patients with relapsed/refractory leukemia and the relatively short follow-up period of our study.

In addition to its retrospective monocentric nature, the small number of patients included with a relatively short follow-up further limit our study. Furthermore, only a few patients received isavuconazole as primary antifungal prophylaxis, and the majority of patients had isavuconazole administered as replacement and not as a first-line treatment. Nevertheless, our data suggest that isavuconazole is a safe antifungal agent in complex and poly-medicated hematological patients, given the relatively low risk of drug interactions and hepatotoxicity and a shortening effect on the QTc interval. With its use increasing overtime, more data on the long-term efficacy and safety profile of this compound are required.

## Data Availability

Data are available upon request.
